# Expression of Ets-1, Ang-2 and maspin in ovarian cancer and their role in tumor angiogenesis

**DOI:** 10.1186/1756-9966-30-31

**Published:** 2011-03-25

**Authors:** Zijing Lin, Yu Liu, Yuhui Sun, Xiuping He

**Affiliations:** 1 Department of Gynaecology and Obstetrics, the 1st affiliated Hospital of Harbin Medical University, Harbin, Heilongjiang Province, PR China

## Abstract

**Background:**

Various angiogenic regulators are involved in angiogenesis cascade. Transcription factor Ets-1 plays important role in angiogenesis, remodeling of extracellular matrix, and tumor metastasis. Ets-1 target genes involved in various stages of new blood vessel formation include angiopoietin, matrix metalloproteinases (MMPs) and the protease inhibitor maspin.

**Methods:**

We used immunohistochemistry (IHC) to detect the expression of Ets-1, angiopoietin-2 (Ang-2) and maspin in ovarian tumor and analyzed the relationship between the expression of these proteins and the clinical manifestation of ovarian cancer.

**Results:**

Ets-1 expression was much stronger in ovarian cancer compared to benign tumors, but had no significant correlation with other pathological parameters of ovarian cancer. However, Ang-2 and maspin expression had no obvious correlation with pathological parameters of ovarian cancer. Ets-1 had a positive correlation with Ang-2 which showed their close relationship in angiogenesis. Although microvessel density (MVD) value had no significant correlation with the expression of Ets-1, Ang-2 or maspin, strong nuclear expression of maspin appeared to be correlated with high grade and MVD.

**Conclusions:**

The expression of Ets-1, Ang2 and maspin showed close relationship with angiogenesis in ovarian cancer and expression of maspin appeared to be correlated with high grade and MVD. The mechanisms underlying the cross-talk of the three factors need further investigations.

## Background

Ovarian cancer is the sixth most common cancer and the sixth most frequent cause of cancer death in women. It is the leading cause of death from gynecologic cancer in women in industrialized countries. The incidence of ovarian carcinoma appears to be increasing in western countries, as evidenced by a 30% rise in incidence and a 18% rise in death rate in the United States. The largely unchanged mortality rate from ovarian carcinoma is due to its late clinical appearance, with two-thirds of the patients being diagnosed as stage III or IV disease [1].

Angiogenesis is the process of formation of blood vessels from pre-existing ones [2]. Without angiogenesis tumor expansion cannot proceed beyond 1-2 mm since tumor proliferation is severely limited by nutrient supply to, and waste removal from, the tumor into the surrounding medium. Therefore, angiogenesis is a crucial factor in the progression of solid tumors and metastases, including epithelial ovarian cancer [3]. Angiogenesis is a complex process which is regulated by the balance between angiogenic activators and inhibitors. Angiogenic factors are produced by various kinds of cells, including angiogenic activators such as transforming growth factors α and β (TGFα, TGFβ), vascular endothelial growth factor (VEGF), fibroblast growth factor-2 (FGF-2), platelet-derived growth factor (PDGF), tumor necrosis factor α (TNF-α), prostaglandin E_2 _and Interleukin 8. The inhibitors include Thrombospondin 1(TSP-1), Angiopoietin (Angs), and endostatin [4]. Accumulating evidence demonstrates that the cooperation between VEGF and Angs plays an important part in angiogenesis [5].

Various angiogenic regulators are involved in the cascade of angiogenesis. Recent evidence suggests that the Ets family of transcription factors play an important role in angiogenesis. Ets-1 is the first member of the family implicated in angiogenesis, remodeling of extracellular matrix (ECM), and tumor cell metastasis [6]. Ets-1 target genes involve in various stages of new blood vessel formation include vascular endothelial growth factor receptor (VEGF-R), matrix metalloproteinases (MMPs) and the protease inhibitors maspin [7]. Immunohistochemical staining demonstrated that Ets-1 was expressed in vascular endothelial cells and cancer cells of ovarian cancer [8]. Furthermore, Ets-1 has been suggested as a prognostic factor for ovarian cancer since there was a significant correlation between microvessel counts, survival rate and Ets-1 level in ovarian cancer [9].

Up to now, four members of Angs family have been identified including Ang-1, Ang-2, Ang-3 and Ang-4, and the receptors of Angs are called "Ties". They play different roles in angiogenesis: Ang-1 and Ang-4 are agonist ligands for Tie2 and induce tyrosin phosphorylation of Tie2, while Ang-2 and Ang-3 are antagonist ligands. They bind to Tie2 without inducing tyrosin phosphorylation, thus blocking the signal transduction which is essential for angiogenesis, recruitment of pericytes and the eventual hematopoiesis [6]. Ang-2 was originally thought to be a competitive factor for Ang-1, however, a recent study revealed that Ang-2 functioned as an agonist when Ang-1 was absent or as a dose-dependent antagonist when Ang-1 was present [10]. In adult, the process of angiogenesis including tumor formation is currently understood as follows: angiogenesis is primarily mediated by VEGF, which promotes the proliferation and migration of endothelial cells and tubal formation; subsequently, Ang-1 leads to vessel maturation and stabilization in physical situations. However, such stabilized vessel can be destabilized by Ang-2, and in the presence of VEGF Ang-2 induces proliferation of vascular endothelial cells, disintegration of basal matrix and promotes cellular migration; in the absence of VEGF, vessel regression would occur due to destabilization effect of endothelial tubal formation mediated by Ang-2 [11]. Therefore, the balance of at least two systems (VEGF-VEGFR and Ang-tie) regulates vessel formation and regression together with natural angiogenic inhibitors [3].

Maspin, a serine protease inhibitor in the serpin superfamily, functions as a tumor suppressor by inhibiting tumor cell motility, invasion, metastasis and angiogenesis [12]. Maspin expression is aberrantly silenced in many human cancers including breast, prostate, and thyroid cancer. Nevertheless, in other malignancies such as pancreatic, lung, and gastric cancer, maspin expression is increased in malignant cells compared to their normal cells of origin [13]. In normal ovarian surface epithelium the expression level of maspin is low while ovarian cancer cell lines expressed high to low level of maspin and maspin expression is correlated with shorter survival in patients with epithelial ovarian cancer [14].

Ets factors have 200 known target genes, including proteases (MMP-1, -3 and- 9, cathepsin) and their inhibitors (TIMP-1), cell cycle molecules (Cyclin D1, p21), regulators of apoptosis(Fas, RARP, Bcl-2, Bcl-XL), adhesion molecules (E-cadherin, integrins), immune response mediators (interleukins, immunoglobulins), and angiogenesis mediators (VEGF receptors Flt-1, flk-1, Tie1 and Tie2) [15]. It is proposed that Ets-1 functions upstream of angiogenesis cascade, since many potent angiogenic factors contain Ets binding sites in their promoter regions. However, the relationship between Ets-1 and some of its target genes involved in angiogenesis has not been fully investigated in ovarian cancer. In the present study, we examined the relationship between the expression of Ets-1 and its targets Ang-2 and maspin in ovarian cancer and their clinical significance.

## Methods

### Patients and tumor samples

All the specimens were obtained from surgical resection at the 1^st ^and 4^th ^affiliated Hospital of Harbin Medical University from 2007 to 2009. The 30 specimens included 21 cases of ovarian cancer and 9 cases of benign ovarian tumor. The patients' information was provided by the pathology departments of the two hospitals, including the age, pathological diagnosis, grade, stage, surgical process and ascites status of each patient. The ovarian tumors were paraffin embedded and fixed with 10% neutral formalin. Clinical stage was determined by criteria of FIGO. The age of the patients ranged from 37 to 69 years old. The study was approved by the Ethics Committee of Harbin Medical University.

### Immunohistochemical staining (IHC)

The ovarian tumors were paraffin embedded and fixed with 10% neutral formalin. The samples were cut as 4-5 μm thick sections. Next the sections were deparaffinized and the antigens were retrieved by steam treatment in a citrate buffer, quenched for 10 min with 3% hydrogen peroxide at room temperature. Then the expression of Ets-1, Ang2, maspin and CD34 was assessed by IHC using specific antibodies as follows: Ets-1 and Maspin (rabbit anti human, 1:150 dilution) were from Santa Cruz Company (USA), Ang-2 (rabbit anti human, 1:100 dilution) was from ABCam company (Shanghai, China), CD34 (clone QBEnd/10) was from Zhongshanjinqiao Biotechnology (Beijing, China). Then the slides were rinsed with PBS and incubated with rabbit and rat serum polyclonal antibody from Zhong Shan biological science and technology ltd (Beijing, China) for 30 min at room temperature. After rinsed with PBS for 30 s, the slides were incubated for 15 min with 0.06% diaminobenzidine and counterstained with Harris modified hematoxylin. As negative controls, the sections were incubated with PBS instead of primary antibodies. CD34 immunostaining was used to determine tumor MVD. The three most hypervascular areas were selected under low power field. Any single endothelial cell or cluster of endothelial cells identified by positive CD34 staining was counted as a single microvessel. MVD was counted as the number of vessels per high-power field (×200). The mean value for the three fields was recorded as the MVD for each tumor sample.

### Evaluation of immunohistochemical staining

Ovarian tumor specimens were categorized into groups by percentage of the cells stained. In addition, staining intensity was scored as 0 (negative), 1+ (weak), 2+ (medium), and 3+ (strong). A combined score based on the staining intensity and the percentage of cells stained was used to assign a final score. We used ocular grid micrometer ruler to calculate total cell count and positive staining cell count according to McCarty [16], and expression rate (X) was determined by the ratio of positive staining cells to total cell count: the expression degree was defined as (-) if X < 10%; 1 + if 10%≦ X < 25%; 2 + if 25%≦X < 50%; 3 + if X ≧ 50%. Each section was given a histoscore calculated by the formula: Σ(*i*+1)×*Pi *(*i *stands for staining density; ranges from 1 to 4, 0 means no staining; *Pi *stands for the percentage of the cells stained) [9].

### Statistical analysis

The data were analyzed using the Statistical Package for the Social Sciences, version 17.0 (SPSS Inc, Chicago, IL, USA). The Mann-Whitney U-test and Kruskal wallis *H *test was used to compare the categorical variables between the groups; Spearman rank correlation was used to evaluate correlation analysis. P values < 0.05 were considered statistically significant.

## Results

### The expression of Ets-1, Ang-2 and maspin in ovarian cancer

**Figure 1:**
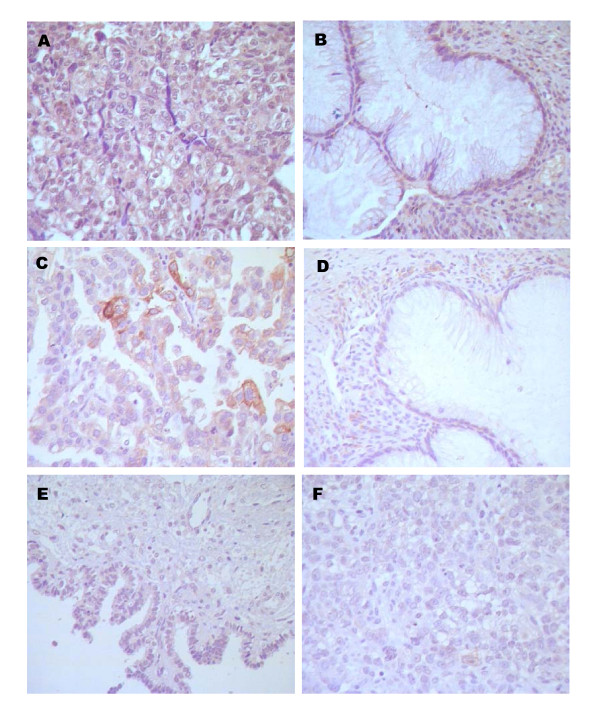
**Immunohistochemical staining for Ets-1, Ang-2 and Maspin in ovarian tumor tissues**. A: Ets-1 expression in ovarian moderately and poorly differentiated serous adenocarcinoma; B: Ets-1 expression in ovarian borderline mucinous cystadenoma; C: Ang-2 expression in left ovarian serous papillary cystadenocarcinoma; D: Ang-2 expression in ovarian borderline mucinous cystadenoma; E: Maspin expression in mucinous cystadenocarcinoma; F: Maspin expression in mucinous cystadenoma. The brown- colored particles deposition region shown in the images stand for positive expression. Ang-2, Angiopoietin-2.

Immunohistochemistry staining showed that Ets-1 was strongly expressed in cancer cells and stroma (Figure 1A) but weakly expressed in benign tumors (Figure 1B). Ang-2 was mainly expressed in tumor stroma and had similar expression pattern in malignant and benign tumors (Figure 1C, D). Maspin expression was predominantly located in the cytoplasma and occasionally in the nucleus of epithelium and cancer cells. The positive expression rate of maspin in benign tumors was 55.56% (5/9) while the rate in ovarian cancer was 52.38% (11/21), there was no significant difference between the two groups (Figure 1E, F).

### The correlation between the expression of Ets-1, Ang-2 and maspin and the clinical manifestation of ovarian cancer

Statistical analysis revealed that Ets-1 expression had no obvious correlation with age, pathological types, grade, stage and ascites formation, but had significant correlation with malignancy of the tumor (Table 1). The expression of Ets-1 was much stronger in ovarian cancer than benign tumors (*p *= 0.022). In contrast, Ang-2 and maspin expression had no significant relationship with the biological behaviors mentioned above. Correlation analysis showed that Ets-1 had a positive correlation with Ang-2 (*p *= 0.0436;*r *= 0.37728), as shown in Table 2, but no significant correlation was found in multiple comparison among the three factors. CD34 staining was used to evaluate MVD and MVD value had no obvious relationship with the expression of the three proteins (Ets-1 and MVD, *p *= 0.1456; Ang-2 and MVD, *p *= 0.2826; maspin and MVD, *p *= 0.6203).

**Table 1:** Correlation analysis of angiogenic factors and clinical manifestation of ovarian tumor

item		n	Ets-1	Maspin	Ang-2
			
			*P*	*p*	*p*
age	< 50	11	0.553	0.582	0.703
	50~	19			
Pathological diagnosis	serous	12	0.651	0.193	0.508
	mucous	5			
	others	4			
grade	Poorly differentiated	10	0.967	0.197	0.160
	Moderately differentiated	7			
	Well differentiated	4			
stage	1	4	0.588	0.916	0.342
	2	7			
	3	7			
	4	1			
ascite	no	8	0.498	0.268	0.916
	yes	13			
Malignant or benign	Benign tumors	9	0.022	0.824	0.209
	Malignant tumors	21			

**Table 2:** Correlation analysis of Ets-1 and Ang-2 expression

Ets-1	Ang-2				Total
		
	-	+	++	+++	
-	5	1	1	0	7
+	4	1	0	1	6
++	4	4	1	1	10
+++	3	1	1	2	7
	
total	16	7	3	4	30

r = 0.37728

*p *= 0.0436

## Discussion

Angiogenesis plays a key role in early embryo development but is rarely found in the adult except in these situations: response to cyclic hormone stimulation of ovary and uterus; damage stress response and other pathological situations such as tumorigenesis and diabetes [17]. Ets-1 expression is upregulated in endothelial cells of neo-vessels during tumor angiogenesis [18]. Thus we hypothesized that Ets-1 expression may be upregulated in ovarian cancer and contribute to ovarian cancer development. Consistent with our hypothesis, in this study we found that Ets-1 had a much stronger expression in ovarian cancer than in benign tumor (p = 0.022), suggesting that Ets-1 is a potential factor that contributes to ovarian cancer angiogenesis. Although a study reported that Ets-1 expression had positive correlation with stage, grade and poor prognosis of ovarian cancer [19], our results showed that Ets-1 expression had no significant relationship with stage and grade (p = 0.867 and 0.588, respectively). The difference may be due to the relative small samples we surveyed.

With regard to Ang-2 expression, it has been reported that Ang-2 and Tie2 expression had no statistical difference between normal ovaries with corpus luteum and ovarian cancer [17]. Our results showed that Ang-2 expression had no obvious difference in ovarian cancer and benign tumor (p = 0.892), consistent with the previous report. We also found that Ang-2 expression tended to be negative in poorly or moderately differentiated ovarian cancer, although P value failed to reach statistical meaning (P = 0.197). Further study employing larger samples will help define the correlation of Ang-2 expression with clinical manifestation of ovarian cancer.

Maspin is widely expressed in mammary epithelium, but is down-regulated in infiltrating cancer and metastatic lesion [20]. It was reported that loss of maspin expression during tumor progression resulted from both the absence of transactivation through the Ets element and the presence of transcription repression through the negative hormonal responsive element (HRE) recognized by androgen receptor [21]. Zhang et al. found that two transcription factors which bound to the promoter of maspin, Ets and Ap1, showed functional incapacitation in metastatic or infiltrative carcinoma [22]. Therefore, we speculated that the reason for negative or weak positive expression of maspin in ovarian cancer was due to the dysfunction of Ets-1 which downregulated maspin expression at transcription level although the expression of Ets-1 was much stronger in ovarian cancer than benign tumors. In this aspect, it is noteworthy that the activity of maspin protein may be modulated by its subcellular localization. Sood et al. found that 4 of 14 benign ovarian neoplasms expressed maspin with mostly nuclear localization; 8 of 10 low malignant potential ovarian tumors had mostly nuclear staining; but only 15 of 57 ovarian cancer had predominant nuclear staining [23]. Our results showed that weak positive expression of maspin in the nucleus appeared only in benign tumors while cytoplasmic strong positive expression was predominantly found in ovarian cancer. In addition, all the 3 cases of cytoplasmic expression of maspin in ovarian cancers were high grade with higher MVD value compared with benign tumors, which was in accordance with previous studies. The mechanisms underlying the localization of maspin and its interaction with Ets-1 warrant further investigations.

In this study we employed IHC to evaluate the expression of Ets-1, Ang-2 and maspin in clinical samples of ovarian cancer. While IHC is an excellent detection technique widely used to understand the distribution and localization of biomarkers and differentially expressed proteins in different parts of tissue samples. Its major disadvantage is that it is impossible to show that the staining corresponds with the protein of interest as in the case of immunoblotting techniques where staining is checked against a molecular weight ladder. For this reason, primary antibodies must be validated by Western Blot before it can be used for IHC. In this study the antibodies for Ets-1, Ang-2 and maspin were commercially derived and validated, and their specificity is warranted.

## Conclusions

In conclusion, our data show that Ets-1 expression was much stronger in ovarian cancer than benign tumors; it had no significant correlation with other biological behaviors, such as grade, stage and ascites. Ang-2 and maspin expression showed no close relationship with biological behaviors mentioned above. Ang-2 had similar expression pattern in ovarian cancer and benign tumors and may be related to vasculature stability during angiogenesis rather than other features of ovarian cancer. Ets-1 had positive correlation with Ang-2 which showed their close relationship in angiogenesis. Maspin expression tended to be determined by subcellular localization and strong nuclear expression of maspin appears to be correlated with high grade and MVD. The connections among the three angiogenic factors Ets-1, Ang-2 and Maspin need future study and the mechanisms by which these factors crosstalk will provide us new therapeutic interventions for ovarian cancer.

## List of abbreviations

(MMPs): matrix metalloproteinases; (IHC): immunohistochemistry; (MVD): microvessel density; (TGFα, TGFβ): transforming growth factors α and β; (VEGF): vascular endothelial growth factors; (FGF-2): fibroblast growth factor-2; (PDGF): platelet-derived growth factor; (TNF-α): tumor necrosis factor α; (TSP-1): Thrombospondin 1; (Angs): Angiopoietin; (ECM): extracellular matrix; (HRE): hormonal responsive element;

## Competing interests

The authors declare that they have no competing interests.

## Authors' contributions

ZJL and YL conceived, coordinated and designed the study and contributed to the acquisition, analysis and interpretation of data and drafted the manuscript. YHS and XPH performed the experiments and were involed in drafting the article. All authors have read and approved the final manuscript.
